# Supplementary material for: Bacterial Succession during Vermicomposting of Silver Wattle (Acacia dealbata Link)
Source: Microorganisms. 2021 Dec 29;10(1):65. doi: 10.3390/microorganisms10010065 (PMC8780150; doi:10.3390/microorganisms10010065)
Supplement: Supplementary file 1 [file microorganisms-10-00065-s001.zip › microorganisms-1503769-supplementary.pdf]

microorganisms

MDPI

Supplementary Materials

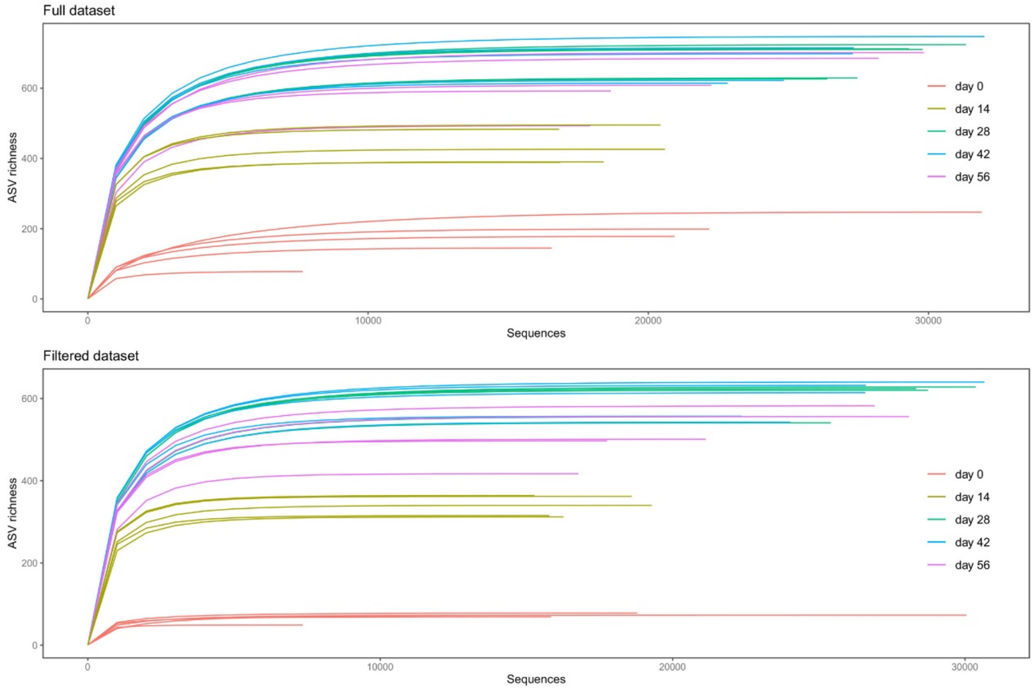

Figure S1. Rarefaction curves of the full and filtered dataset indicating the number of amplicon sequence variants (ASVs) found in each sample during vermicomposting of the silver wattle.

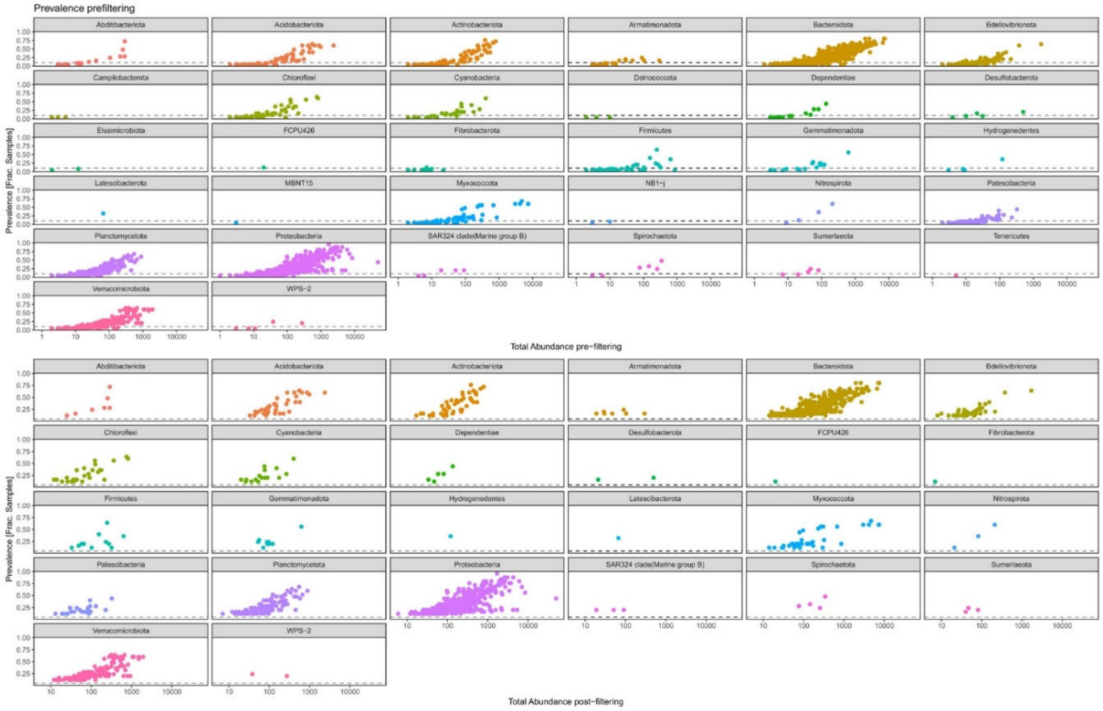

Figure S2. Taxa prevalence versus total counts before and after doing a prevalence filtering using ten percent of all samples as the prevalence threshold. Each point is a different bacterial ASV.

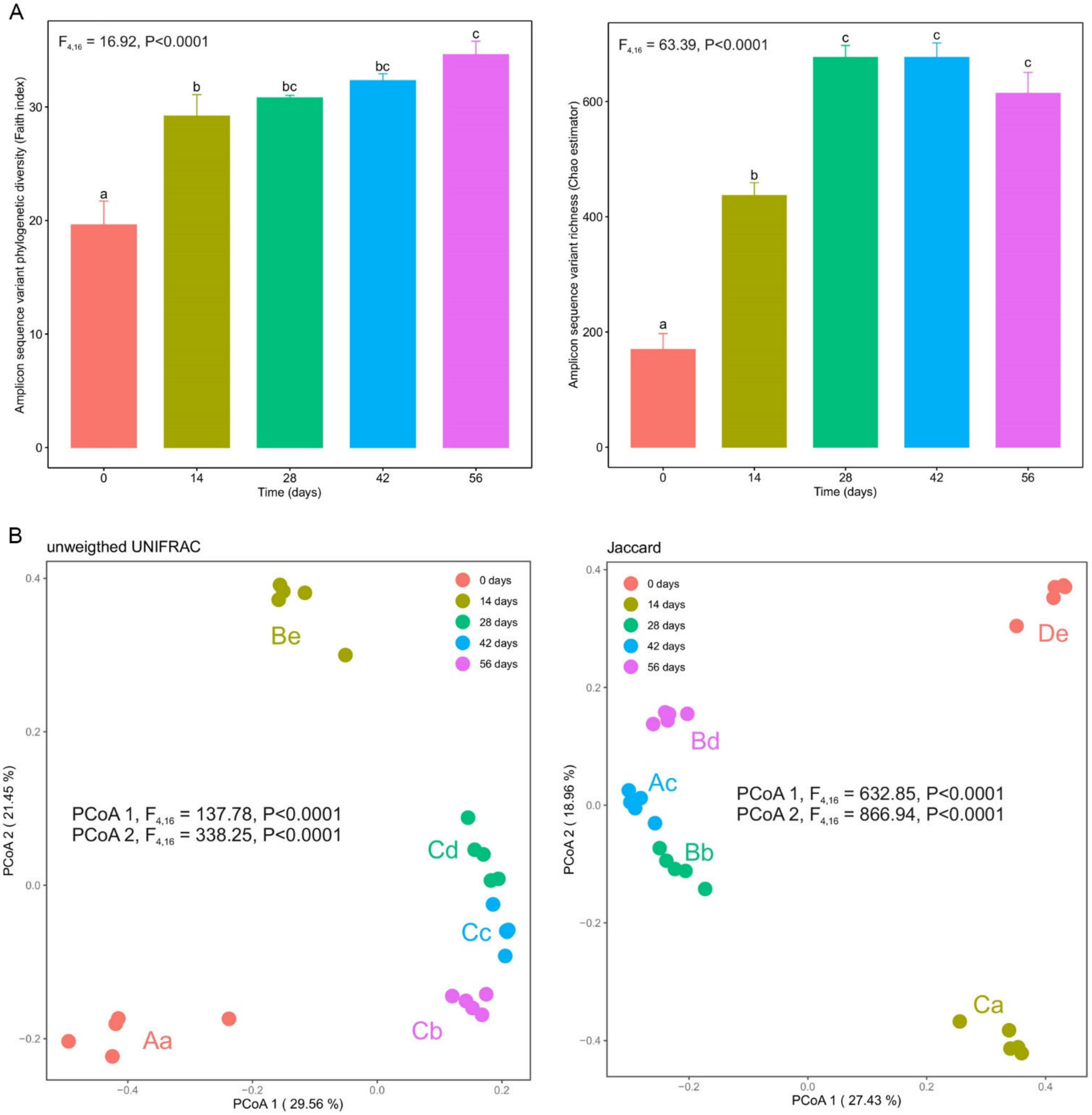

Figure S3. Change in bacterial α- and β-diversity during vermicomposting of the silver wattle. (**A**) α-Diversity is shown in terms of richness (Chao1 estimator) and phylogenetic diversity (Faith's PD index). Letters indicate significant differences between time points (Tukey HSD test). (**B**) β-diversity is shown by principal coordinate analysis (PCoA) of unweighted UniFrac and Jaccard distances. Capital and lower case letters indicate significant differences between the time points in PCoA1 and PCoA2 scores, respectively (Tukey HSD test, FDR corrected).

Table S1. Differential abundance in bacterial taxa during vermicomposting of silver wattle (*Acacia dealbata*) analyzed using negative binomial models as implemented in the package DESeq2.

baseMean	log2FoldChange	lfcSE	stat	pvalue	padj	Phylum	Class	Order	Family	Genus	ASV	Comparison
11.6639	22.37758698	4.652888148	4.809397146	1.51E-06	0.000259565	Bacteroidota	Bacteroidia	Flavobacteriales	Crocinitomicaceae	unclassified	ASV304	day 0 vs. day 14
22.6331	21.53157189	3.268241307	6.588121825	4.45E-11	6.01E-08	Verrucomicrobiota	Verrucomicrobiae	Verrucomicrobiales	Rubritaleaceae	Luteolibacter	ASV159	day 0 vs. day 14
5.0203	21.47486958	4.653610889	4.61466807	3.94E-06	0.000553346	Bacteroidota	Bacteroidia	Flavobacteriales	Flavobacteriaceae	Flavobacterium	ASV605	day 0 vs. day 14
4.26875	21.26818736	4.653834072	4.570035595	4.88E-06	0.000626596	Verrucomicrobiota	Verrucomicrobiae	Verrucomicrobiales	Rubritaleaceae	Luteolibacter	ASV668	day 0 vs. day 14
8.9198	21.25949719	4.537546145	4.685240988	2.80E-06	0.000423307	Myxococcota	Myxococcia	Myxococcales	Myxococcaceae	Corallococcus	ASV392	day 0 vs. day 14
2.67549	20.73129315	4.654721731	4.453820089	8.44E-06	0.001007193	Chloroflexi	Chloroflexia	Chloroflexales	Chloroflexaceae	FFCH7168	ASV886	day 0 vs. day 14
3.06629	20.59072286	4.654419261	4.423908054	9.69E-06	0.001143967	Bacteroidota	Bacteroidia	Cytophagales	Spirosomaceae	Leadbetterella	ASV803	day 0 vs. day 14
2.40493	20.58729372	4.654989346	4.422629611	9.75E-06	0.001143967	Proteobacteria	Alphaproteobacteria	Sphingomonadales	Sphingomonadaceae	Novosphingobium	ASV956	day 0 vs. day 14
26.8853	18.97776915	2.758469911	6.879817348	5.99E-12	1.16E-08	Bacteroidota	Bacteroidia	Chitinophagales	Chitinophagaceae	Chitinophaga	ASV198	day 0 vs. day 14
239.332	10.91120898	1.448836459	7.531014918	5.03E-14	1.36E-10	Proteobacteria	Gammaproteobacteria	Burkholderiales	Comamonadaceae	unclassified	ASV9	day 0 vs. day 14
57.8103	10.12287103	1.798493618	5.628527636	1.82E-08	6.81E-06	Bacteroidota	Bacteroidia	Chitinophagales	Chitinophagaceae	Ferruginibacter	ASV60	day 0 vs. day 14
88.1879	10.04688968	1.591135141	6.314290613	2.71E-10	2.82E-07	Proteobacteria	Alphaproteobacteria	Sphingomonadales	Sphingomonadaceae	Novosphingobium	ASV33	day 0 vs. day 14
46.2191	10.02596697	2.482655929	4.03840373	5.38E-05	0.004624763	Bacteroidota	Bacteroidia	Flavobacteriales	Flavobacteriaceae	Flavobacterium	ASV72	day 0 vs. day 14
82.0538	9.760162743	1.633314938	5.975677144	2.29E-09	1.63E-06	Proteobacteria	Gammaproteobacteria	Burkholderiales	Comamonadaceae	Hydrogenophaga	ASV41	day 0 vs. day 14
39.9443	9.709124282	1.985708767	4.889500637	1.01E-06	0.00018684	Proteobacteria	Alphaproteobacteria	Caulobacterales	Caulobacteraceae	Asticcacaulis	ASV81	day 0 vs. day 14
45.133	9.678246061	1.823082248	5.308727058	1.10E-07	3.10E-05	Proteobacteria	Alphaproteobacteria	Caulobacterales	Caulobacteraceae	Brevundimonas	ASV77	day 0 vs. day 14
43.4936	9.661043817	1.903249757	5.076077789	3.85E-07	8.66E-05	Bacteroidota	Bacteroidia	Cytophagales	Spirosomaceae	Persicitalea	ASV78	day 0 vs. day 14
33.3808	9.516813074	2.307587767	4.12413916	3.72E-05	0.003303089	Bacteroidota	Bacteroidia	Chitinophagales	Chitinophagaceae	unclassified	ASV96	day 0 vs. day 14
157.076	9.494378765	1.609800701	5.897859755	3.68E-09	2.16E-06	Proteobacteria	Alphaproteobacteria	Sphingomonadales	Sphingomonadaceae	Novosphingobium	ASV18	day 0 vs. day 14

67.8938	9.317963757	1.763557601	5.283617475	1.27E-07	3.49E-05	Proteobacteria	Alphaproteobacteria	Caulobacterales	Hyphomonadaceae	Hirschia	ASV57	day 0 vs. day 14
49.8355	9.294754639	1.853548364	5.014573571	5.32E-07	0.000108654	Bacteroidota	Bacteroidia	Sphingobacteriales	Sphingobacteriaceae	Sphingobacterium	ASV75	day 0 vs. day 14
57.7921	9.261675774	1.834738813	5.047953261	4.47E-07	9.47E-05	Proteobacteria	Gammaproteobacteria	Xanthomonadales	Xanthomonadaceae	Stenotrophomonas	ASV68	day 0 vs. day 14
26.9827	9.179108574	2.497175972	3.675795649	0.000237109	0.015837021	Proteobacteria	Alphaproteobacteria	Sphingomonadales	Sphingomonadaceae	Novosphingobium	ASV126	day 0 vs. day 14
24.5047	9.135210525	2.482676095	3.679582103	0.000233616	0.015759767	Proteobacteria	Gammaproteobacteria	Burkholderiales	Comamonadaceae	Pseudorhodoferax	ASV141	day 0 vs. day 14
78.7606	9.031485133	1.445711319	6.247087518	4.18E-10	3.76E-07	Proteobacteria	Gammaproteobacteria	Xanthomonadales	Rhodanobacteraceae	Dokdonella	ASV52	day 0 vs. day 14
91.1179	8.927165305	1.89152608	4.71955708	2.36E-06	0.000372518	Bacteroidota	Bacteroidia	Flavobacteriales	Flavobacteriaceae	Flavobacterium	ASV42	day 0 vs. day 14
25.5863	8.814457429	2.100045095	4.197270549	2.70E-05	0.002563985	Bacteroidota	Bacteroidia	Flavobacteriales	Crocinitomicaceae	Fluviicola	ASV146	day 0 vs. day 14
114.265	8.773686618	1.478613134	5.93372696	2.96E-09	1.82E-06	Bacteroidota	Bacteroidia	Cytophagales	Microscillaceae	Ohtaekwangia	ASV31	day 0 vs. day 14
339.35	8.751857554	1.419623785	6.164913301	7.05E-10	5.95E-07	Bacteroidota	Bacteroidia	Flavobacteriales	Flavobacteriaceae	Flavobacterium	ASV3	day 0 vs. day 14
35.5437	8.713839711	1.743588386	4.997647254	5.80E-07	0.000112112	Proteobacteria	Gammaproteobacteria	Xanthomonadales	unclassified	unclassified	ASV108	day 0 vs. day 14
17.9165	8.637688513	2.110511331	4.092699426	4.26E-05	0.003711434	Bacteroidota	Bacteroidia	Chitinophagales	Chitinophagaceae	unclassified	ASV194	day 0 vs. day 14
26.5026	8.60735155	1.879191343	4.58034866	4.64E-06	0.000607548	Proteobacteria	Gammaproteobacteria	Pseudomonadales	Moraxellaceae	Cavicella	ASV149	day 0 vs. day 14
83.9261	8.569311839	1.465357817	5.847931295	4.98E-09	2.59E-06	Bacteroidota	Bacteroidia	Cytophagales	Microscillaceae	unclassified	ASV49	day 0 vs. day 14
84.8164	8.543137446	1.845051796	4.630296809	3.65E-06	0.000524095	Proteobacteria	Gammaproteobacteria	Burkholderiales	Comamonadaceae	unclassified	ASV48	day 0 vs. day 14
66.7854	8.447763424	2.368395389	3.566872096	0.000361268	0.022358825	Proteobacteria	Gammaproteobacteria	Burkholderiales	Comamonadaceae	Rhizobacter	ASV66	day 0 vs. day 14
18.478	8.378014011	2.287325588	3.662799059	0.000249474	0.01658082	Proteobacteria	Gammaproteobacteria	Xanthomonadales	Xanthomonadaceae	Luteimonas	ASV206	day 0 vs. day 14
26.5684	8.334764499	2.376940237	3.506509911	0.000454025	0.027469502	Bacteroidota	Bacteroidia	Flavobacteriales	Flavobacteriaceae	Flavobacterium	ASV153	day 0 vs. day 14
166.776	8.319941375	1.930946746	4.308736836	1.64E-05	0.001717245	Bacteroidota	Bacteroidia	Flavobacteriales	Flavobacteriaceae	Flavobacterium	ASV17	day 0 vs. day 14

30.946	8.199425926	1.813674656	4.520891273	6.16E-06	0.000769292	Bacteroidota	Bacteroidia	Sphingobacteriales	Sphingobacteriaceae	unclassified	ASV140	day 0 vs. day 14
100.245	8.189989559	1.413057772	5.79593398	6.79E-09	3.27E-06	Proteobacteria	Gammaproteobacteria	Burkholderiales	Alcaligenaceae	Achromobacter	ASV35	day 0 vs. day 14
121.708	8.152070906	1.911372225	4.265035768	2.00E-05	0.002012565	Bacteroidota	Bacteroidia	Flavobacteriales	Flavobacteriaceae	Flavobacterium	ASV27	day 0 vs. day 14
20.8428	8.119904397	2.130498855	3.811269074	0.000138255	0.010249114	Bacteroidota	Bacteroidia	Sphingobacteriales	Sphingobacteriaceae	Pedobacter	ASV204	day 0 vs. day 14
16.3509	8.06866892	2.045579015	3.944442556	8.00E-05	0.006540417	Proteobacteria	Gammaproteobacteria	Oceanospirillales	Pseudohongiellaceae	Pseudohongiella	ASV240	day 0 vs. day 14
18.0791	8.058720291	1.752379617	4.598729756	4.25E-06	0.000585215	Bacteroidota	Bacteroidia	Chitinophagales	Chitinophagaceae	Niabella	ASV216	day 0 vs. day 14
37.0859	8.040881135	1.457387685	5.517324743	3.44E-08	1.16E-05	Proteobacteria	Gammaproteobacteria	Burkholderiales	Methylophilaceae	Methylophilus	ASV116	day 0 vs. day 14
20.6074	7.991181077	1.927153	4.14662514	3.37E-05	0.003057676	Bacteroidota	Bacteroidia	Chitinophagales	Chitinophagaceae	Niabella	ASV203	day 0 vs. day 14
26.8003	7.945246035	1.934754823	4.106590634	4.02E-05	0.003517925	Bacteroidota	Bacteroidia	Sphingobacteriales	Sphingobacteriaceae	Pedobacter	ASV161	day 0 vs. day 14
117.885	7.940459764	1.468738571	5.406312545	6.43E-08	1.89E-05	Proteobacteria	Gammaproteobacteria	Pseudomonadales	Moraxellaceae	unclassified	ASV30	day 0 vs. day 14
28.1138	7.858977226	1.843005608	4.26421775	2.01E-05	0.002012565	Proteobacteria	Gammaproteobacteria	Pseudomonadales	Pseudomonadaceae	Pseudomonas	ASV157	day 0 vs. day 14
30.7511	7.70399477	1.99815482	3.855554481	0.000115468	0.008752185	Chloroflexi	Chloroflexia	Chloroflexales	Chloroflexaceae	FFCH7168	ASV150	day 0 vs. day 14
153.221	7.684860634	1.623086084	4.734721535	2.19E-06	0.000356573	Proteobacteria	Alphaproteobacteria	Sphingomonadales	Sphingomonadaceae	Novosphingobium	ASV21	day 0 vs. day 14
52.9824	7.426497907	1.484717087	5.001961633	5.67E-07	0.000112112	Proteobacteria	Alphaproteobacteria	Sphingomonadales	Sphingomonadaceae	Novosphingobium	ASV83	day 0 vs. day 14
34.9969	7.405343183	2.202900671	3.361632814	0.000774831	0.041984007	Chloroflexi	Chloroflexia	Chloroflexales	Chloroflexaceae	FFCH7168	ASV131	day 0 vs. day 14
326.821	7.374546031	1.411924102	5.22304706	1.76E-07	4.57E-05	Bacteroidota	Bacteroidia	Flavobacteriales	Flavobacteriaceae	Flavobacterium	ASV6	day 0 vs. day 14
30.0752	7.292499364	1.726407211	4.224089958	2.40E-05	0.002345537	Proteobacteria	Alphaproteobacteria	Caulobacterales	Caulobacteraceae	unclassified	ASV156	day 0 vs. day 14
32.361	7.259992261	1.619261858	4.483519589	7.34E-06	0.000900552	Proteobacteria	Alphaproteobacteria	Caulobacterales	Caulobacteraceae	Phenylobacterium	ASV148	day 0 vs. day 14
27.2748	7.022049886	2.085213007	3.367545599	0.000758405	0.041259669	Acidobacteriota	Blastocatellia	Blastocatellales	Blastocatellaceae	Blastocatella	ASV172	day 0 vs. day 14

27.8081	6.971053553	2.040429876	3.416463185	0.000634402	0.036115432	Verrucomicrobiota	Verrucomicrobiae	Verrucomicrobiales	Rubritaleaceae	Luteolibacter	ASV166	day 0 vs. day 14
14.8962	6.878325364	1.591445059	4.322062721	1.55E-05	0.001629341	Actinobacteriota	Thermoleophilia	Solirubrobacterales	Solirubrobacteraceae	Patulibacter	ASV306	day 0 vs. day 14
12.6392	6.835820807	1.769300097	3.863573409	0.00011174	0.008565909	Proteobacteria	Alphaproteobacteria	Rhizobiales	Rhizobiaceae	Neorhizobium	ASV358	day 0 vs. day 14
19.8846	6.817978846	1.745079679	3.906972803	9.35E-05	0.007374023	Proteobacteria	Alphaproteobacteria	Rhizobiales	Rhizobiaceae	Shinella	ASV243	day 0 vs. day 14
34.4255	6.597968739	1.775539786	3.716035424	0.000202373	0.014002151	Actinobacteriota	Actinobacteria	Micrococcales	Microbacteriaceae	unclassified	ASV143	day 0 vs. day 14
322.211	6.550672377	1.927976209	3.397693575	0.000679565	0.038044356	Proteobacteria	Alphaproteobacteria	Sphingomonadales	Sphingomonadaceae	Novosphingobium	ASV7	day 0 vs. day 14
40.196	6.428640002	1.687907171	3.80864547	0.00013973	0.010301852	Proteobacteria	Alphaproteobacteria	Caulobacterales	Caulobacteraceae	Brevundimonas	ASV88	day 0 vs. day 14
167.863	6.373790004	1.760199212	3.621061731	0.000293396	0.018940218	Bacteroidota	Bacteroidia	Flavobacteriales	Flavobacteriaceae	Flavobacterium	ASV19	day 0 vs. day 14
16.6996	6.176866697	1.862177632	3.317012616	0.000909855	0.048140254	Verrucomicrobiota	Verrucomicrobiae	Verrucomicrobiales	Verrucomicrobiaceae	unclassified	ASV305	day 0 vs. day 14
77.3662	5.98885143	1.421754964	4.212295072	2.53E-05	0.00245369	Proteobacteria	Gammaproteobacteria	Cellvibrionales	Cellvibrionaceae	Cellvibrio	ASV46	day 0 vs. day 14
41.4158	5.565279247	1.619474236	3.436472852	0.000589341	0.034207214	Proteobacteria	Alphaproteobacteria	Reyranellales	Reyranellaceae	Reyranella	ASV114	day 0 vs. day 14
2060.3	-8.87197114	2.253554775	-3.93687841	8.25E-05	0.00666912	Proteobacteria	Gammaproteobacteria	Pseudomonadales	Pseudomonadaceae	Pseudomonas	ASV1	day 0 vs. day 14
80.6744	-11.5967316	3.390398803	-3.42046241	0.000625148	0.035891462	Proteobacteria	Alphaproteobacteria	Rhizobiales	Beijerinckiaceae	1174-901-12	ASV39	day 0 vs. day 14
81.1113	-11.6045261	3.401054395	-3.41203779	0.000644792	0.036552648	Proteobacteria	Alphaproteobacteria	Rhizobiales	Beijerinckiaceae	unclassified	ASV38	day 0 vs. day 14
154.799	-12.5369151	3.390000929	-3.69820404	0.00021713	0.014870672	Proteobacteria	Alphaproteobacteria	Rhizobiales	Beijerinckiaceae	1174-901-12	ASV13	day 0 vs. day 14
197.395	-12.887584	3.402738788	-3.78741503	0.000152223	0.011041876	Proteobacteria	Alphaproteobacteria	Rhizobiales	Beijerinckiaceae	1174-901-12	ASV10	day 0 vs. day 14
2.64697	-21.4526	4.655544079	-4.6079684	4.07E-06	0.000565582	Firmicutes	Clostridia	Clostridiales	Clostridiaceae	Clostridium sensu stricto 1	ASV972	day 0 vs. day 14
2.61346	-21.4987394	4.65558165	-4.61784177	3.88E-06	0.000550689	Proteobacteria	Alphaproteobacteria	Rhizobiales	Beijerinckiaceae	1174-901-12	ASV977	day 0 vs. day 14

3.08255	-21.7254973	4.655091494	-4.6670398	3.06E-06	0.00045305	Firmicutes	Bacilli	Erysipelotrichales	Erysipelotrichaceae	Turicibacter	ASV869	day 0 vs. day 14
3.58514	-21.9528538	4.654708498	-4.71626823	2.40E-06	0.000372518	Firmicutes	Clostridia	Peptostreptococcales-Tissierellales	Peptostreptococcaceae	Intestinibacter	ASV786	day 0 vs. day 14
5.49842	-22.1459834	4.653891154	-4.75859505	1.95E-06	0.000324716	Proteobacteria	Gammaproteobacteria	Enterobacterales	unclassified	unclassified	ASV638	day 0 vs. day 14
8.58589	-22.647402	3.944070789	-5.74213882	9.35E-09	4.07E-06	Firmicutes	Clostridia	Clostridiales	Clostridiaceae	Clostridium sensu stricto 1	ASV483	day 0 vs. day 14
7.76371	-22.718201	4.653444198	-4.88201856	1.05E-06	0.00019145	Verrucomicrobiota	Verrucomicrobiae	Pedosphaerales	Pedosphaeraceae	unclassified	ASV630	day 0 vs. day 14
9.60228	-22.9777921	4.173484943	-5.50566072	3.68E-08	1.18E-05	Firmicutes	Clostridia	Clostridiales	Clostridiaceae	Clostridium sensu stricto 1	ASV418	day 0 vs. day 14
11.1228	-23.512336	4.554946533	-5.16193458	2.44E-07	5.98E-05	Firmicutes	Clostridia	Peptostreptococcales-Tissierellales	Peptostreptococcaceae	Romboutsia	ASV366	day 0 vs. day 14
13.3937	-23.6442614	4.255111629	-5.55667241	2.75E-08	9.76E-06	Bacteroidota	Bacteroidia	Chitinophagales	Chitinophagaceae	unclassified	ASV412	day 0 vs. day 14
28.4801	-24.8572563	4.652653983	-5.34259724	9.16E-08	2.63E-05	Firmicutes	Bacilli	Lactobacillales	Enterococcaceae	Enterococcus	ASV128	day 0 vs. day 14
21.3799	42.61738251	3.29932478	12.91700131	3.61E-38	2.43E-34	Proteobacteria	Alphaproteobacteria	Rhizobiales	Xanthobacteraceae	unclassified	ASV245	day 14 vs. day 28
21.9844	41.33615203	3.091580164	13.37055804	8.99E-41	1.21E-36	Proteobacteria	Gammaproteobacteria	Burkholderiales	Chromobacteriaceae	Chromobacterium	ASV228	day 14 vs. day 28
25.7165	30.45460006	4.663588818	6.53029271	6.56E-11	7.44E-08	Bacteroidota	Bacteroidia	Flavobacteriales	Weeksellaceae	Elizabethkingia	ASV234	day 14 vs. day 28
8.58589	19.28786084	3.962723612	4.867324276	1.13E-06	0.000203494	Firmicutes	Clostridia	Clostridiales	Clostridiaceae	Clostridium sensu stricto 1	ASV483	day 14 vs. day 28
11.1228	19.18463598	4.573922374	4.194351022	2.74E-05	0.002563985	Firmicutes	Clostridia	Peptostreptococcales-Tissierellales	Peptostreptococcaceae	Romboutsia	ASV366	day 14 vs. day 28
9.60228	19.13658811	4.193069472	4.563861447	5.02E-06	0.000639231	Firmicutes	Clostridia	Clostridiales	Clostridiaceae	Clostridium sensu stricto 1	ASV418	day 14 vs. day 28
335.262	12.26719733	1.722205092	7.122959623	1.06E-12	2.38E-09	Myxococcota	Polyangia	Polyangiales	Sandaracinaceae	unclassified	ASV5	day 14 vs. day 28
108.927	11.85671061	1.787218225	6.6341706	3.26E-11	4.89E-08	Acidobacteriota	Blastocatellia	Blastocatellales	Blastocatellaceae	unclassified	ASV34	day 14 vs. day 28

337.606	11.72854377	1.721425622	6.813273614	9.54E-12	1.61E-08	Myxococcota	Polyangia	Polyangiales	Sandaracinaceae	unclassified	ASV4	day 14 vs. day 28
89.6453	10.90194519	1.734740523	6.284481768	3.29E-10	3.17E-07	Verrucomicrobiota	Verrucomicrobiae	Verrucomicrobiales	Verrucomicrobiaceae	unclassified	ASV51	day 14 vs. day 28
96.5513	10.64927653	1.740743469	6.117659911	9.50E-10	7.54E-07	Bacteroidota	Bacteroidia	Cytophagales	Microscillaceae	Chryseolinea	ASV47	day 14 vs. day 28
99.8262	10.59847516	1.948891731	5.438206232	5.38E-08	1.65E-05	Bacteroidota	Bacteroidia	Flavobacteriales	Flavobacteriaceae	Flavobacterium	ASV44	day 14 vs. day 28
53.1923	10.33236069	1.733603711	5.960047632	2.52E-09	1.70E-06	Verrucomicrobiota	Verrucomicrobiae	Verrucomicrobiales	Rubritaleaceae	Luteolibacter	ASV89	day 14 vs. day 28
62.7253	10.31333498	1.721210992	5.991906296	2.07E-09	1.55E-06	Bacteroidota	Bacteroidia	Chitinophagales	Chitinophagaceae	Terrimonas	ASV76	day 14 vs. day 28
73.2086	10.26080773	2.328199479	4.407185819	1.05E-05	0.001187323	Bacteroidota	Bacteroidia	Flavobacteriales	Flavobacteriaceae	Flavobacterium	ASV64	day 14 vs. day 28
98.1304	10.19585839	1.805857685	5.645992191	1.64E-08	6.33E-06	Bacteroidota	Bacteroidia	Chitinophagales	Chitinophagaceae	unclassified	ASV45	day 14 vs. day 28
121.611	10.17252567	1.714088507	5.934656015	2.94E-09	1.82E-06	Bacteroidota	Bacteroidia	Chitinophagales	Chitinophagaceae	Parafilimonas	ASV28	day 14 vs. day 28
80.2377	10.08307766	1.731167999	5.824436258	5.73E-09	2.86E-06	Bacteroidota	Bacteroidia	Flavobacteriales	Flavobacteriaceae	Flavobacterium	ASV56	day 14 vs. day 28
140.77	10.0567499	1.814148957	5.543508357	2.96E-08	1.03E-05	Myxococcota	Polyangia	Polyangiales	Sandaracinaceae	unclassified	ASV25	day 14 vs. day 28
37.4344	9.948246949	2.285830385	4.35213698	1.35E-05	0.001478822	Bacteroidota	Bacteroidia	Cytophagales	Microscillaceae	Ohtaekwangia	ASV134	day 14 vs. day 28
70.9751	9.913323699	1.713728083	5.784653817	7.27E-09	3.38E-06	Proteobacteria	Gammaproteobacteria	Burkholderiales	Comamonadaceae	unclassified	ASV67	day 14 vs. day 28
73.4551	9.902799295	1.715971215	5.770958865	7.88E-09	3.54E-06	Proteobacteria	Gammaproteobacteria	Pseudomonadales	Moraxellaceae	Cavicella	ASV63	day 14 vs. day 28
51.8222	9.853043828	1.969226315	5.003510135	5.63E-07	0.000112112	Bacteroidota	Bacteroidia	Flavobacteriales	Flavobacteriaceae	Flavobacterium	ASV93	day 14 vs. day 28
41.9555	9.813611111	1.910740516	5.136025028	2.81E-07	6.55E-05	Bacteroidota	Bacteroidia	Flavobacteriales	Flavobacteriaceae	Flavobacterium	ASV117	day 14 vs. day 28
90.5941	9.806353238	1.718537829	5.706219014	1.16E-08	4.58E-06	Bacteroidota	Bacteroidia	Cytophagales	Microscillaceae	unclassified	ASV50	day 14 vs. day 28

158.039	9.773850052	1.711250768	5.711524129	1.12E-08	4.58E-06	Proteobacteria	Gammaproteobacteria	Steroidobacterales	Steroidobacteraceae	unclassified	ASV20	day 14 vs. day 28
178.006	9.733554038	1.702212753	5.718177132	1.08E-08	4.54E-06	Bacteroidota	Bacteroidia	Chitinophagales	Chitinophagaceae	unclassified	ASV16	day 14 vs. day 28
30.1147	9.697321242	2.303941671	4.209013345	2.56E-05	0.002471814	Verrucomicrobiota	Verrucomicrobiae	Verrucomicrobiales	Rubritaleaceae	unclassified	ASV171	day 14 vs. day 28
44.9699	9.681606985	1.94472979	4.978381591	6.41E-07	0.00012015	Acidobacteriota	Vicinamibacteria	Vicinamibacterales	unclassified	unclassified	ASV107	day 14 vs. day 28
39.698	9.637857897	1.994890422	4.831271829	1.36E-06	0.000240839	Proteobacteria	Gammaproteobacteria	Cellvibrionales	Spongiibacteraceae	BD1-7 clade	ASV124	day 14 vs. day 28
26.5621	9.615203021	2.357534986	4.078498549	4.53E-05	0.003920246	Planctomycetota	Planctomycetes	Pirellulales	Pirellulaceae	Pir4 lineage	ASV197	day 14 vs. day 28
190.306	9.501596299	1.738176548	5.466416118	4.59E-08	1.44E-05	Myxococcota	Polyangia	Polyangiales	Sandaracinaceae	unclassified	ASV14	day 14 vs. day 28
51.5005	9.446898942	1.961341805	4.816549016	1.46E-06	0.000255931	Bacteroidota	Bacteroidia	Flavobacteriales	Flavobacteriaceae	Flavobacterium	ASV92	day 14 vs. day 28
73.8281	9.440331614	1.963214385	4.808609638	1.52E-06	0.000259565	Proteobacteria	Alphaproteobacteria	Sphingomonadales	Sphingomonadaceae	Novosphingobium	ASV62	day 14 vs. day 28
34.002	9.422107784	2.156558328	4.369048434	1.25E-05	0.001380045	Proteobacteria	Gammaproteobacteria	Burkholderiales	Comamonadaceae	unclassified	ASV152	day 14 vs. day 28
141.858	9.417326568	1.794693093	5.247318667	1.54E-07	4.16E-05	Bacteroidota	Bacteroidia	Chitinophagales	Chitinophagaceae	Chitinophaga	ASV24	day 14 vs. day 28
68.5557	9.336765889	1.722295656	5.421116785	5.92E-08	1.78E-05	Verrucomicrobiota	Verrucomicrobiae	Pedosphaerales	Pedosphaeraceae	unclassified	ASV71	day 14 vs. day 28
26.375	9.317565231	2.663842725	3.497791045	0.000469129	0.028256617	Acidobacteriota	Vicinamibacteria	Vicinamibacterales	Vicinamibacteraceae	Luteitalea	ASV199	day 14 vs. day 28
27.9862	9.250643827	2.300497354	4.021149517	5.79E-05	0.004914383	Bacteroidota	Bacteroidia	Flavobacteriales	Flavobacteriaceae	Flavobacterium	ASV192	day 14 vs. day 28
33.3479	9.170742569	1.917138499	4.783557669	1.72E-06	0.000290448	Bacteroidota	Bacteroidia	Flavobacteriales	Flavobacteriaceae	Flavobacterium	ASV158	day 14 vs. day 28
42.262	9.140264764	1.967157375	4.646432908	3.38E-06	0.000489954	Proteobacteria	Gammaproteobacteria	Burkholderiales	Comamonadaceae	unclassified	ASV111	day 14 vs. day 28
27.4867	9.137278807	2.484472328	3.677754308	0.000235296	0.015794127	Proteobacteria	Alphaproteobacteria	Rhizobiales	unclassified	unclassified	ASV195	day 14 vs. day 28

31.798	9.112816335	2.330110978	3.910893696	9.20E-05	0.007341184	Bacteroidota	Bacteroidia	Chitinophagales	Chitinophagaceae	Terrimonas	ASV162	day 14 vs. day 28
76.2048	9.080689346	1.760103068	5.159180453	2.48E-07	5.98E-05	Bacteroidota	Bacteroidia	Sphingobacteriales	env.OPS 17	unclassified	ASV61	day 14 vs. day 28
48.2634	9.035122433	1.759359887	5.135460061	2.81E-07	6.55E-05	Bacteroidota	Bacteroidia	Flavobacteriales	Crocinitomicaceae	Fluviicola	ASV100	day 14 vs. day 28
24.9457	9.008707817	1.965223271	4.584063271	4.56E-06	0.000603206	Acidobacteriota	Acidobacteriae	Paludibaculum	unclassified	unclassified	ASV208	day 14 vs. day 28
46.3363	9.004811863	1.784278529	5.046752353	4.49E-07	9.47E-05	Proteobacteria	Gammaproteobacteria	Burkholderiales	SC-I-84	unclassified	ASV105	day 14 vs. day 28
24.1655	8.99302483	2.300868686	3.908534583	9.29E-05	0.007369621	Verrucomicrobiota	Verrucomicrobiae	Verrucomicrobiales	Rubritaleaceae	Luteolibacter	ASV221	day 14 vs. day 28
28.087	8.986144975	2.310458356	3.889334318	0.00010052	0.007839364	Proteobacteria	Gammaproteobacteria	Burkholderiales	Comamonadaceae	unclassified	ASV187	day 14 vs. day 28
37.4859	8.941807535	1.950495894	4.584376498	4.55E-06	0.000603206	Proteobacteria	Gammaproteobacteria	Xanthomonadales	Xanthomonadaceae	Thermomonas	ASV135	day 14 vs. day 28
24.3184	8.919925899	2.288369124	3.897940156	9.70E-05	0.007609989	Proteobacteria	Gammaproteobacteria	Xanthomonadales	Xanthomonadaceae	Pseudoxanthomonas	ASV217	day 14 vs. day 28
49.0309	8.918467325	1.72721307	5.163501526	2.42E-07	5.98E-05	Proteobacteria	Alphaproteobacteria	Reyranellales	Reyranellaceae	Reyranella	ASV95	day 14 vs. day 28
21.1982	8.917114329	2.338187028	3.813687366	0.000136909	0.010205367	Bacteroidota	Bacteroidia	Sphingobacteriales	Sphingobacteriaceae	Mucilaginibacter	ASV259	day 14 vs. day 28
21.2513	8.894730903	2.386823169	3.726598191	0.000194081	0.013638268	Proteobacteria	Alphaproteobacteria	Sphingomonadales	Sphingomonadaceae	Novosphingobium	ASV258	day 14 vs. day 28
20.853	8.789909814	2.577527138	3.410210385	0.000649128	0.036644489	Bacteroidota	Bacteroidia	Cytophagales	Microscillaceae	unclassified	ASV275	day 14 vs. day 28
22.3027	8.766439092	2.321384517	3.77638389	0.000159122	0.011480587	Bacteroidota	Bacteroidia	Flavobacteriales	Flavobacteriaceae	Flavobacterium	ASV247	day 14 vs. day 28
24.908	8.744204127	1.743704424	5.014728416	5.31E-07	0.000108654	Verrucomicrobiota	Verrucomicrobiae	Chthoniobacterales	Chthoniobacteraceae	Chthoniobacter	ASV210	day 14 vs. day 28
39.8692	8.743477365	1.730152739	5.053586985	4.34E-07	9.44E-05	Acidobacteriota	Acidobacteriae	unclassified	unclassified	unclassified	ASV121	day 14 vs. day 28
190.298	8.72851519	1.680296778	5.194627108	2.05E-07	5.22E-05	Bacteroidota	Bacteroidia	Flavobacteriales	Flavobacteriaceae	Flavobacterium	ASV15	day 14 vs. day 28

50.9376	8.722652169	1.746134843	4.995405828	5.87E-07	0.000112112	Proteobacteria	Gammaproteobacteria	Xanthomonadales	Xanthomonadaceae	Arenimonas	ASV90	day 14 vs. day 28
18.6022	8.710752718	2.308458937	3.773405963	0.000161034	0.011556754	Bacteroidota	Bacteroidia	Chitinophagales	Chitinophagaceae	Edaphobaculum	ASV299	day 14 vs. day 28
39.9924	8.644120367	1.730738425	4.994469554	5.90E-07	0.000112112	Planctomycetota	Planctomycetes	Planctomycetales	unclassified	unclassified	ASV119	day 14 vs. day 28
101.056	8.61568831	1.952768129	4.412038573	1.02E-05	0.00118086	Proteobacteria	Alphaproteobacteria	Sphingomonadales	Sphingomonadaceae	Novosphingobium	ASV40	day 14 vs. day 28
14.492	8.604735559	2.32419593	3.702241902	0.000213703	0.014710597	Bacteroidota	Bacteroidia	Sphingobacteriales	env.OPS 17	unclassified	ASV375	day 14 vs. day 28
15.0746	8.602217207	2.224870336	3.866390355	0.000110458	0.008516003	Bacteroidota	Bacteroidia	Cytophagales	Cyclobacteriaceae	Algoriphagus	ASV365	day 14 vs. day 28
14.1847	8.589282845	2.32839037	3.688935909	0.000225194	0.015267922	Proteobacteria	Gammaproteobacteria	Xanthomonadales	Xanthomonadaceae	Pseudoxanthomonas	ASV380	day 14 vs. day 28
23.6805	8.578382662	1.974991736	4.343503067	1.40E-05	0.001513571	Actinobacteriota	Actinobacteria	Streptomycetales	Streptomycetaceae	Streptomyces	ASV218	day 14 vs. day 28
21.2972	8.573053111	1.944418383	4.409057837	1.04E-05	0.001187082	Bacteroidota	Bacteroidia	Sphingobacteriales	NS11-12 marine group	unclassified	ASV249	day 14 vs. day 28
14.9512	8.571745802	2.221963828	3.857734178	0.000114443	0.008723532	Verrucomicrobiota	Verrucomicrobiae	Verrucomicrobiales	Verrucomicrobiaceae	unclassified	ASV362	day 14 vs. day 28
52.5617	8.464003833	1.897114967	4.461513393	8.14E-06	0.000980373	Proteobacteria	Gammaproteobacteria	Burkholderiales	Comamonadaceae	unclassified	ASV87	day 14 vs. day 28
13.3899	8.454432187	2.499385974	3.382603677	0.000718022	0.039703069	Proteobacteria	Alphaproteobacteria	Sphingomonadales	Sphingomonadaceae	Sphingorhabdus	ASV396	day 14 vs. day 28
28.571	8.33822251	1.938365742	4.301676576	1.70E-05	0.001759259	Proteobacteria	Alphaproteobacteria	Caulobacterales	Caulobacteraceae	unclassified	ASV174	day 14 vs. day 28
40.0297	8.285685514	1.756430412	4.717343458	2.39E-06	0.000372518	Bacteroidota	Bacteroidia	Chitinophagales	Chitinophagaceae	Ferruginibacter	ASV122	day 14 vs. day 28
16.4183	8.2787095	1.952874744	4.239242443	2.24E-05	0.002224943	Verrucomicrobiota	Verrucomicrobiae	Chthoniobacterales	Terrimicrobiaceae	Terrimicrobium	ASV314	day 14 vs. day 28
16.4844	8.278157369	2.226162735	3.718576921	0.000200348	0.0139335	Bacteroidota	Bacteroidia	Cytophagales	Spirosomaceae	Dyadobacter	ASV333	day 14 vs. day 28
25.4667	8.277514976	1.742948438	4.749145067	2.04E-06	0.000336113	Acidobacteriota	Vicinamibacteria	Vicinamibacterales	Vicinamibacteraceae	unclassified	ASV205	day 14 vs. day 28

15.5301	8.266068555	2.081950605	3.970348064	7.18E-05	0.005904207	Proteobacteria	Alphaproteobacteria	Rhodobacterales	Rhodobacteraceae	unclassified	ASV350	day 14 vs. day 28
16.8527	8.25245208	2.477492202	3.330969951	0.000865439	0.045970507	Bacteroidota	Bacteroidia	Sphingobacteriales	Sphingobacteriaceae	Mucilaginibacter	ASV323	day 14 vs. day 28
23.9259	8.189843687	1.95603139	4.186969458	2.83E-05	0.002630511	Bacteroidota	Bacteroidia	Chitinophagales	Chitinophagaceae	Taibaiella	ASV223	day 14 vs. day 28
15.9667	8.183022416	1.95025695	4.195868866	2.72E-05	0.002563985	Proteobacteria	Gammaproteobacteria	Xanthomonadales	Rhodanobacteraceae	Tahibacter	ASV341	day 14 vs. day 28
27.4203	8.153001689	1.740888196	4.68324256	2.82E-06	0.000423307	Proteobacteria	Alphaproteobacteria	Caulobacterales	Caulobacteraceae	Phenylobacterium	ASV191	day 14 vs. day 28
16.9069	8.135831075	2.249959352	3.615990248	0.000299202	0.019222993	Verrucomicrobiota	Verrucomicrobiae	Chthoniobacterales	Xiphinematobacteraceae	Candidatus Xiphinematobacter	ASV318	day 14 vs. day 28
39.0925	8.097822234	1.728480023	4.684938284	2.80E-06	0.000423307	Bacteroidota	Bacteroidia	Flavobacteriales	Flavobacteriaceae	Flavobacterium	ASV127	day 14 vs. day 28
16.0065	8.096524918	2.130452553	3.800377955	0.000144476	0.010593828	Verrucomicrobiota	Verrucomicrobiae	Verrucomicrobiales	Rubritaleaceae	Luteolibacter	ASV338	day 14 vs. day 28
13.9441	8.08615763	2.337096075	3.459916653	0.000540343	0.031696967	Bacteroidota	Bacteroidia	Chitinophagales	Chitinophagaceae	unclassified	ASV385	day 14 vs. day 28
27.6307	8.077501707	1.948054623	4.146445183	3.38E-05	0.003057676	Bacteroidota	Bacteroidia	Flavobacteriales	Flavobacteriaceae	Flavobacterium	ASV182	day 14 vs. day 28
31.5082	8.04972967	1.948786788	4.130636414	3.62E-05	0.003232365	Myxococcota	Polyangia	Polyangiales	Sandaracinaceae	unclassified	ASV163	day 14 vs. day 28
20.6628	8.038233314	1.929187464	4.166641897	3.09E-05	0.00283717	Bacteroidota	Bacteroidia	Chitinophagales	Chitinophagaceae	Edaphobaculum	ASV272	day 14 vs. day 28
31.23	8.033754881	1.749225512	4.592749664	4.37E-06	0.000596161	Proteobacteria	Gammaproteobacteria	Pseudomonadales	Moraxellaceae	unclassified	ASV165	day 14 vs. day 28
22.101	8.020428275	1.751757669	4.578503304	4.68E-06	0.000607548	Actinobacteriota	Acidimicrobiia	Microtrichales	unclassified	unclassified	ASV236	day 14 vs. day 28
21.5261	8.010690116	2.14594668	3.732939961	0.000189258	0.013368927	Acidobacteriota	Blastocatellia	Blastocatellales	Blastocatellaceae	unclassified	ASV250	day 14 vs. day 28
48.4764	7.987921682	1.814816178	4.401504558	1.08E-05	0.001198701	Proteobacteria	Alphaproteobacteria	Rhizobiales	Xanthobacteraceae	Rhodopseudomonas	ASV98	day 14 vs. day 28
15.7232	7.93218915	2.105530736	3.767311022	0.000165015	0.011717828	Bacteroidota	Bacteroidia	Chitinophagales	Chitinophagaceae	Ferruginibacter	ASV347	day 14 vs. day 28

17.7681	7.929909867	1.755192523	4.517971539	6.24E-06	0.000772818	Proteobacteria	Alphaproteobacteria	Caulobacterales	Caulobacteraceae	Phenylobacterium	ASV303	day 14 vs. day 28
47.5399	7.924936891	1.726606978	4.589890456	4.43E-06	0.000598341	Verrucomicrobiota	Verrucomicrobiae	Opitutales	Opitutaceae	Lacunisphaera	ASV97	day 14 vs. day 28
10.6504	7.91652604	2.325500638	3.404224412	0.000663522	0.037301005	Planctomycetota	Planctomycetes	Gemmatales	Gemmataceae	Zavarzinella	ASV489	day 14 vs. day 28
14.2769	7.889390192	2.299468915	3.430906188	0.000601445	0.034678194	Bacteroidota	Bacteroidia	Cytophagales	Cytophagaceae	unclassified	ASV378	day 14 vs. day 28
19.319	7.871193768	2.324671627	3.3859379	0.000709354	0.039385221	Proteobacteria	Gammaproteobacteria	Legionellales	Legionellaceae	Legionella	ASV288	day 14 vs. day 28
104.961	7.841239819	2.172563496	3.609210885	0.00030713	0.019638844	Proteobacteria	Gammaproteobacteria	Xanthomonadales	Xanthomonadaceae	Thermomonas	ASV36	day 14 vs. day 28
23.2824	7.788307459	1.744477398	4.464550512	8.02E-06	0.000975277	Bacteroidota	Bacteroidia	Flavobacteriales	Flavobacteriaceae	Flavobacterium	ASV224	day 14 vs. day 28
10.9612	7.778089681	2.126396416	3.657873773	0.000254316	0.01673773	Bacteroidota	Bacteroidia	Flavobacteriales	Flavobacteriaceae	Flavobacterium	ASV478	day 14 vs. day 28
15.6886	7.762580662	1.924923767	4.032669134	5.51E-05	0.004709126	Planctomycetota	Planctomycetes	Pirellulales	Pirellulaceae	unclassified	ASV348	day 14 vs. day 28
23.4804	7.747165685	1.755525935	4.413016937	1.02E-05	0.00118086	Proteobacteria	Alphaproteobacteria	Rhodobacterales	Rhodobacteraceae	unclassified	ASV220	day 14 vs. day 28
67.306	7.697191941	1.953496576	3.940212661	8.14E-05	0.006616721	Verrucomicrobiota	Verrucomicrobiae	Pedosphaerales	Pedosphaeraceae	unclassified	ASV70	day 14 vs. day 28
14.9852	7.592086589	2.160298452	3.514369315	0.0004408	0.02691071	Proteobacteria	Alphaproteobacteria	Sphingomonadales	Sphingomonadaceae	Altererythrobacter	ASV359	day 14 vs. day 28
15.0196	7.56722863	1.768356125	4.27924473	1.88E-05	0.001931401	Bacteroidota	Bacteroidia	Chitinophagales	Chitinophagaceae	unclassified	ASV356	day 14 vs. day 28
33.98	7.54068176	1.740896488	4.331493466	1.48E-05	0.001573373	Proteobacteria	Gammaproteobacteria	Xanthomonadales	Rhodanobacteraceae	Rhodanobacter	ASV151	day 14 vs. day 28
19.0778	7.534505703	1.739193344	4.332184072	1.48E-05	0.001573373	Acidobacteriota	Blastocatellia	Blastocatellales	Blastocatellaceae	unclassified	ASV290	day 14 vs. day 28
288.177	7.499302355	1.611187511	4.654518672	3.25E-06	0.000476236	Bacteroidota	Bacteroidia	Chitinophagales	Chitinophagaceae	Edaphobaculum	ASV8	day 14 vs. day 28
11.0963	7.499298214	1.935839517	3.873925575	0.000107096	0.008304259	Proteobacteria	Gammaproteobacteria	Xanthomonadales	Xanthomonadaceae	Xanthomonas	ASV468	day 14 vs. day 28

20.1603	7.431141792	1.770708697	4.196704858	2.71E-05	0.002563985	Verrucomicrobiota	Verrucomicrobiae	Opitutales	Opitutaceae	unclassified	ASV270	day 14 vs. day 28
12.7367	7.429020948	1.796064554	4.136277247	3.53E-05	0.003174989	Verrucomicrobiota	Verrucomicrobiae	Verrucomicrobiales	Verrucomicrobiaceae	Roseimicrobium	ASV413	day 14 vs. day 28
9.03816	7.40047899	2.217841566	3.336793351	0.000847509	0.045196033	Planctomycetota	Planctomycetes	Pirellulales	Pirellulaceae	unclassified	ASV545	day 14 vs. day 28
89.135	7.383650211	1.944653126	3.796898332	0.000146518	0.010685507	Bacteroidota	Bacteroidia	Sphingobacteriales	Sphingobacteriaceae	Mucilaginibacter	ASV53	day 14 vs. day 28
17.5391	7.372817177	1.729344325	4.263359859	2.01E-05	0.002012565	Bdellovibrionota	Oligoflexia	0319-6G20	unclassified	unclassified	ASV309	day 14 vs. day 28
20.5583	7.352373365	1.739340852	4.227103249	2.37E-05	0.002331251	Planctomycetota	Planctomycetes	Gemmatales	Gemmataceae	Gemmata	ASV260	day 14 vs. day 28
41.179	7.350205321	2.150560892	3.417808511	0.000631275	0.036089673	Bacteroidota	Bacteroidia	Chitinophagales	Chitinophagaceae	unclassified	ASV118	day 14 vs. day 28
28.427	7.321002609	1.940927206	3.771909934	0.000162003	0.011564767	Gemmatimonadota	Gemmatimonadetes	Gemmatimonadales	Gemmatimonadaceae	unclassified	ASV181	day 14 vs. day 28
9.53783	7.286199661	1.827530333	3.986910384	6.69E-05	0.005609596	Nitrospirota	Nitrospiria	Nitrospirales	Nitrospiraceae	Nitrospira	ASV523	day 14 vs. day 28
42.1593	7.227995612	1.731298393	4.174898816	2.98E-05	0.002754942	Proteobacteria	Gammaproteobacteria	Burkholderiales	Comamonadaceae	unclassified	ASV110	day 14 vs. day 28
16.5134	7.190000022	1.745395253	4.119410781	3.80E-05	0.003349565	Planctomycetota	Planctomycetes	Gemmatales	Gemmataceae	Gemmata	ASV321	day 14 vs. day 28
18.1846	7.164423998	1.804213483	3.970940283	7.16E-05	0.005904207	Cyanobacteria	Vampirivibrionia	Obscuribacterales	Obscuribacteraceae	unclassified	ASV296	day 14 vs. day 28
12.3007	7.160179069	1.959493915	3.654096098	0.00025809	0.016903616	Myxococcota	Polyangia	Polyangiales	Polyangiaceae	Pajaroellobacter	ASV429	day 14 vs. day 28
41.1789	7.147275435	1.935805421	3.692145584	0.00022237	0.015152613	Proteobacteria	Gammaproteobacteria	Cellvibrionales	Cellvibrionaceae	Cellvibrio	ASV115	day 14 vs. day 28
8.75341	7.111379285	1.785927026	3.981898019	6.84E-05	0.005693883	Proteobacteria	Alphaproteobacteria	Dongiales	Dongiaceae	Dongia	ASV549	day 14 vs. day 28
7.81482	7.075058862	2.083206445	3.396235106	0.000683196	0.038089616	Bacteroidota	Bacteroidia	Cytophagales	Microscillaceae	OLB12	ASV595	day 14 vs. day 28

26.895	7.032894419	1.930648062	3.642763566	0.000269727	0.017580439	Verrucomicrobiota	Verrucomicrobiae	Pedosphaerales	Pedosphaeraceae	unclassified	ASV186	day 14 vs. day 28
56.0851	7.03011918	1.836240145	3.828540183	0.000128906	0.009716169	Bacteroidota	Bacteroidia	Chitinophagales	Chitinophagaceae	Aurantisolimonas	ASV84	day 14 vs. day 28
218.128	6.99789525	1.641121951	4.264092164	2.01E-05	0.002012565	Myxococcota	Polyangia	Polyangiales	Sandaracinaceae	unclassified	ASV11	day 14 vs. day 28
12.8914	6.96772948	1.779727812	3.915053432	9.04E-05	0.007258711	Acidobacteriota	Acidobacteriae	unclassified	unclassified	unclassified	ASV415	day 14 vs. day 28
82.6969	6.946992499	1.938873042	3.583005359	0.000339664	0.021236872	Bacteroidota	Bacteroidia	Chitinophagales	Chitinophagaceae	Sediminibacterium	ASV54	day 14 vs. day 28
7.09186	6.868471309	1.973599291	3.480175201	0.000501086	0.029651983	Bacteroidota	Bacteroidia	Sphingobacteriales	Sphingobacteriaceae	Solitalea	ASV632	day 14 vs. day 28
18.8553	6.847772551	1.940716798	3.528475952	0.00041796	0.025749385	Proteobacteria	Gammaproteobacteria	Burkholderiales	Comamonadaceae	unclassified	ASV293	day 14 vs. day 28
20.279	6.777556095	1.972610938	3.435830129	0.000590741	0.034207214	Proteobacteria	Gammaproteobacteria	Xanthomonadales	Rhodanobacteraceae	Dokdonella	ASV277	day 14 vs. day 28
15.7298	6.706145005	1.756266256	3.818410212	0.000134314	0.010067614	Actinobacteriota	Thermoleophilia	Solirubrobacterales	Solirubrobacteraceae	Conexibacter	ASV342	day 14 vs. day 28
9.85927	6.52140883	1.945415867	3.35219268	0.000801742	0.043268411	Verrucomicrobiota	Verrucomicrobiae	Opitutales	Opitutaceae	IMCC26134	ASV518	day 14 vs. day 28
11.0497	6.503448412	1.787007733	3.639295059	0.000273385	0.01773325	Proteobacteria	Gammaproteobacteria	Burkholderiales	Nitrosomonadaceae	Ellin6067	ASV461	day 14 vs. day 28
14.3461	6.463189069	1.935172987	3.339850811	0.000838234	0.044878786	Myxococcota	Polyangia	Polyangiales	BIrii41	unclassified	ASV384	day 14 vs. day 28
149.908	6.343786592	1.876108367	3.381354032	0.000721295	0.039721298	Bacteroidota	Bacteroidia	Flavobacteriales	Flavobacteriaceae	Flavobacterium	ASV22	day 14 vs. day 28
12.6793	6.228514001	1.785434599	3.488514228	0.000485713	0.029125508	Planctomycetota	Planctomycetes	Pirellulales	Pirellulaceae	Pirellula	ASV409	day 14 vs. day 28
46.6467	6.095305573	1.697359576	3.591051454	0.000329347	0.020800794	Bacteroidota	Bacteroidia	Chitinophagales	Chitinophagaceae	Chitinophaga	ASV101	day 14 vs. day 28
23.0274	5.895414788	1.692776172	3.482690083	0.000496403	0.029551705	Proteobacteria	Alphaproteobacteria	Reyranellales	Reyranellaceae	Reyranella	ASV229	day 14 vs. day 28
167.863	5.083860909	1.505582968	3.376672702	0.000733683	0.04023923	Bacteroidota	Bacteroidia	Flavobacteriales	Flavobacteriaceae	Flavobacterium	ASV19	day 14 vs. day 28

326.821	4.744320149	1.077787249	4.401907847	1.07E-05	0.001198701	Bacteroidota	Bacteroidia	Flavobacteriales	Flavobacteriaceae	Flavobacterium	ASV6	day 14 vs. day 28
2.40493	-23.5527233	4.655148248	-5.05950017	4.20E-07	9.30E-05	Proteobacteria	Alphaproteobacteria	Sphingomonadales	Sphingomonadaceae	Novosphingobium	ASV956	day 14 vs. day 28
2.67549	-23.6943197	4.65488063	-5.09020995	3.58E-07	8.18E-05	Chloroflexi	Chloroflexia	Chloroflexales	Chloroflexaceae	FFCH7168	ASV886	day 14 vs. day 28
4.26875	-24.3973041	4.653992961	-5.24223056	1.59E-07	4.20E-05	Verrucomicrobiota	Verrucomicrobiae	Verrucomicrobiales	Rubritaleaceae	Luteolibacter	ASV668	day 14 vs. day 28
3.06629	-25.6598265	4.654578157	-5.51281462	3.53E-08	1.16E-05	Bacteroidota	Bacteroidia	Cytophagales	Spirosomaceae	Leadbetterella	ASV803	day 14 vs. day 28
5.0203	-26.0631614	4.653769776	-5.60044064	2.14E-08	7.80E-06	Bacteroidota	Bacteroidia	Flavobacteriales	Flavobacteriaceae	Flavobacterium	ASV605	day 14 vs. day 28
11.6639	-27.3592887	4.653047027	-5.87986508	4.11E-09	2.31E-06	Bacteroidota	Bacteroidia	Flavobacteriales	Crocinitomicaceae	unclassified	ASV304	day 14 vs. day 28
26.3932	8.376884199	2.322612422	3.606664685	0.000310158	0.019738926	Bacteroidota	Bacteroidia	Cytophagales	Cytophagaceae	Cytophaga	ASV196	day 28 vs. day 42
23.9816	8.221439751	2.294726605	3.582753489	0.000339991	0.021236872	Verrucomicrobiota	Verrucomicrobiae	Pedosphaerales	Pedosphaeraceae	unclassified	ASV213	day 28 vs. day 42
14.0121	7.887053358	2.339698596	3.370969822	0.000749041	0.040915207	Bacteroidota	Bacteroidia	Cytophagales	Cytophagaceae	Cytophaga	ASV381	day 28 vs. day 42
32.5075	-8.06375764	2.335000045	-3.45342933	0.000553507	0.03232866	Bacteroidota	Bacteroidia	Cytophagales	Spirosomaceae	Leadbetterella	ASV104	day 28 vs. day 42
11.1228	-18.2733655	4.573922374	-3.9951193	6.47E-05	0.005452597	Firmicutes	Clostridia	Peptostreptococcales-Tissierellales	Peptostreptococcaceae	Romboutsia	ASV366	day 28 vs. day 42
8.9198	-26.6631158	4.559794817	-5.84743762	4.99E-09	2.59E-06	Myxococcota	Myxococcia	Myxococcales	Myxococcaceae	Corallococcus	ASV392	day 28 vs. day 42
25.7165	-30.4485332	4.663588818	-6.52899181	6.62E-11	7.44E-08	Bacteroidota	Bacteroidia	Flavobacteriales	Weeksellaceae	Elizabethkingia	ASV234	day 28 vs. day 42
22.6331	37.32960719	3.27136166	11.41103035	3.68E-30	1.66E-26	Verrucomicrobiota	Verrucomicrobiae	Verrucomicrobiales	Rubritaleaceae	Luteolibacter	ASV159	day 42 vs. day 56
27.9862	8.22948921	2.301021042	3.576451088	0.00034829	0.021654999	Bacteroidota	Bacteroidia	Flavobacteriales	Flavobacteriaceae	Flavobacterium	ASV192	day 42 vs. day 56
22.3027	8.084351785	2.321582045	3.482259781	0.000497201	0.029551705	Bacteroidota	Bacteroidia	Flavobacteriales	Flavobacteriaceae	Flavobacterium	ASV247	day 42 vs. day 56

24.1655	8.07560296	2.301343724	3.509081619	0.000449657	0.027327796	Verrucomicrobiota	Verrucomicrobiae	Verrucomicrobiales	Rubritaleaceae	Luteolibacter	ASV221	day 42 vs. day 56
14.9725	7.992690711	2.307265091	3.464140616	0.000531928	0.03133964	Verrucomicrobiota	Verrucomicrobiae	Chthoniobacterales	Chthoniobacteraceae	Chthoniobacter	ASV370	day 42 vs. day 56
35.5437	7.941595092	1.745349978	4.55014478	5.36E-06	0.000675975	Proteobacteria	Gammaproteobacteria	Xanthomonadales	unclassified	unclassified	ASV108	day 42 vs. day 56
57.8103	7.830506589	1.801142571	4.347521798	1.38E-05	0.001498095	Bacteroidota	Bacteroidia	Chitinophagales	Chitinophagaceae	Ferruginibacter	ASV60	day 42 vs. day 56
30.1147	7.716508286	2.305584799	3.346876805	0.000817275	0.043930985	Verrucomicrobiota	Verrucomicrobiae	Verrucomicrobiales	Rubritaleaceae	unclassified	ASV171	day 42 vs. day 56
20.8428	7.655090075	2.131984966	3.590592897	0.000329927	0.020800794	Bacteroidota	Bacteroidia	Sphingobacteriales	Sphingobacteriaceae	Pedobacter	ASV204	day 42 vs. day 56
26.8003	6.816468745	1.938715716	3.515971263	0.000438148	0.026870447	Bacteroidota	Bacteroidia	Sphingobacteriales	Sphingobacteriaceae	Pedobacter	ASV161	day 42 vs. day 56
12.6392	6.592960096	1.772390764	3.719811811	0.000199371	0.0139335	Proteobacteria	Alphaproteobacteria	Rhizobiales	Rhizobiaceae	Neorhizobium	ASV358	day 42 vs. day 56
18.0791	6.435034498	1.758817317	3.658728191	0.00025347	0.01673773	Bacteroidota	Bacteroidia	Chitinophagales	Chitinophagaceae	Niabella	ASV216	day 42 vs. day 56
13.3937	-20.1187531	4.260051322	-4.72265509	2.33E-06	0.000372518	Bacteroidota	Bacteroidia	Chitinophagales	Chitinophagaceae	unclassified	ASV412	day 42 vs. day 56
25.7165	-35.206965	4.652900345	-7.56667076	3.83E-14	1.29E-10	Bacteroidota	Bacteroidia	Flavobacteriales	Weeksellaceae	Elizabethkingia	ASV234	day 42 vs. day 56
